# Chronic *Strongyloides stercoralis* infection increases presence of the *Ruminococcus torques* group in the gut and alters the microbial proteome

**DOI:** 10.1038/s41598-023-31118-5

**Published:** 2023-03-14

**Authors:** Na T. D. Tran, Apisit Chaidee, Achirawit Surapinit, Manachai Yingklang, Sitiruk Roytrakul, Sawanya Charoenlappanit, Porntip Pinlaor, Nuttanan Hongsrichan, Sirirat Anutrakulchai, Ubon Cha’on, Somchai Pinlaor

**Affiliations:** 1 Department of Parasitology, Faculty of Medicine, Khon Kaen University, Khon Kaen, 40002 Thailand; 2 Faculty of Public Health, Burapha University, Chonburi, 20131 Thailand; 3 Functional Proteomics Technology Laboratory, National Center for Genetic Engineering and Biotechnology, National Science and Technology Development Agency, Pathum Thani, Thailand; 4 Centre for Research and Development of Medical Diagnostic Laboratories, Faculty of Associated Medical Sciences, Khon Kaen University, Khon Kaen, 40002 Thailand; 5 Department of Medicine, Faculty of Medicine, Khon Kaen University, Khon Kaen, 40002 Thailand; 6 Department of Biochemistry, Faculty of Medicine, Khon Kaen University, Khon Kaen, 40002 Thailand; 7 Chronic Kidney Disease Prevention in Northeastern Thailand, Khon Kaen University, Khon Kaen, 40002 Thailand

**Keywords:** Infectious diseases, Parasitic infection, Computational biology and bioinformatics, Microbiology, Diseases, Medical research

## Abstract

We explored the impact of chronic *Strongyloides stercoralis* infection on the gut microbiome and microbial activity in a longitudinal study. At baseline (time-point T0), 42 fecal samples from matched individuals (21 positive for strongyloidiasis and 21 negative) were subjected to microbiome 16S-rRNA sequencing. Those positive at T0 (untreated then because of COVID19 lockdowns) were retested one year later (T1). Persistent infection in these individuals indicated chronic strongyloidiasis: they were treated with ivermectin and retested four months later (T2). Fecal samples at T1 and T2 were subjected to 16S-rRNA sequencing and LC–MS/MS to determine microbial diversity and proteomes. No significant alteration of indices of gut microbial diversity was found in chronic strongyloidiasis. However, the *Ruminococcus torques* group was highly over-represented in chronic infection. Metaproteome data revealed enrichment of *Ruminococcus torques* mucin-degrader enzymes in infection, possibly influencing the ability of the host to expel parasites. Metaproteomics indicated an increase in carbohydrate metabolism and *Bacteroidaceae* accounted for this change in chronic infection. STITCH interaction networks explored highly expressed microbial proteins before treatment and short-chain fatty acids involved in the synthesis of acetate. In conclusion, our data indicate that chronic *S. stercoralis* infection increases *Ruminococcus torques* group and alters the microbial proteome.

## Introduction

The nematode *Strongyloides stercoralis* is a neglected soil-transmitted helminth (STH) affecting approximately 600 million people around the world^1^. Adult female *S. stercoralis* inhabits in the small intestine. Infection usually occurs when filariform larvae penetrate through the skin to initiate the parasitic cycle^2^. The South-East Asian, African, Latin American and Western Pacific regions have the highest prevalence of strongyloidiasis. This can be a chronic, lifelong infection because *S. stercoralis* has the unique ability to undertake autoinfection of its host^3^. In chronic infection, there is reduced fecal excretion of larvae: the parasite persists with low worm burden and generally causes no symptoms (but a variety of gastrointestinal manifestations, pulmonary and cutaneous manifestations may occur)^2,4^. Chronic strongyloidiasis can remain unnoticed for decades. In Northeast Thailand, the prevalence of strongyloidiasis has remained high (around 25%) over a long time period^5–7^, predominantly among those over the age of 60. Hyperinfection or disseminated infections of *S. stercoralis* typically affect immunosuppressed hosts, such as those undergoing corticosteroid treatment, infected with human T-cell lymphotropic virus type 1 (HTLV-1) or elderly individuals, and can cause fatal disease^8–10^.

Helminths have a range of effects on the human gut microbiome^11^. The gut microflora has important roles in human health via fermentation of nondigestible components of food, protection of the host from pathogenic bacteria, and regulation of immunity. Helminth infections (*Trichuris trichiura*, *Ascaris* spp. and hookworm) might have either positive or negative roles in maintaining gut homeostasis via modulation of the gut microbiota in both human and animal hosts^12^. In humans*, Strongyloides* spp*.* adults in the digestive tract interact with the host gut microbiota, which can have an impact on gut homeostasis^13–15^. A longitudinal study in non-endemic areas indicated an increase of pathogenic bacteria after treatment^16^, while a cross-sectional study in Thailand showed the ability of infection to increase pathogenic microorganisms^14^. Thus, reported effects of *S. stercor*alis infection appear to vary depending on study design and geographic location. A cross-sectional study has limited explanatory power to clarify the association between infection and alteration of gut microbiome. Longitudinal studies of *S. stercoralis* infections and follow-up after treatment provide an ideal chance to assess the effects and interactions of chronic *S. stercoralis* colonization on the composition of the human gut microbiota.

Among the “-omics” approaches available for the investigation of microbial communities, 16S-rRNA next-generation sequencing (16S-rRNA NGS) is a useful tool to explore complex and diverse microbial communities. However, these sequences cannot determine cause-and-effect relationships between host and infection. Determination of the functional activity of microbial communities provides direct insight at the molecular level through identification and quantification of proteins produced by the microbes. Liquid chromatography with tandem mass spectrometry (LC–MS/MS) has emerged as a new tool that allows reliable, fast, and cost-effective identification of activities of microorganisms through identification of their proteins in host fecal material^17,18^.

In this study, we explore the impact of natural chronic infections by *S. stercoralis* on the fecal microbiota of a cohort of participants in an endemic area for parasites in Northeast Thailand. Analyses of bacterial 16S-rRNA high-throughput sequencing data was applied to compare the fecal microbial profiles of positive individuals compared to negative individuals at baseline. After 1 year, we undertook 16S-rRNA NGS and fecal proteomic microbiota profiling (using LC–MS/MS) of a subset of baseline subjects with chronic infection, both prior to and following treatment with anti-helminthic treatment. This study will contribute to knowledge of the effects of chronic helminth infection before and after treatment on the gut microbial community in endemic areas. Our work provides the first metaproteomic data to detect the effects of chronic helminth infection on the structure and function of the gut microbial community in individuals.

## Results

### Study population characteristics and biochemical parameters

Supplementary Table S1 displays the demographic and biochemical information of participants at the baseline (T0). Sample were coded as Pos (positive) or Neg (negative) for *S. stercoralis* infection. All samples were matched at T0 between Pos and Neg groups in terms of sex, age, body-mass index, and all biochemical test parameters were within the normal range. As a result, there were no differences in blood pressure, eGFR, HbA1c, glucose, or LDL cholesterol levels between the two groups. Only eosinophilia, typically seen in parasitic infections and allergic reactions, showed a significant increase in the Pos group.

### Gut microbiome results

The gut microbiome was evaluated from 16S-rRNA sequences obtained from fecal samples. These data were treated as two separate sets for the comparisons done. The first set of comparisons was between Pos and Neg samples at baseline (T0). The second set was collected 1 + years after baseline and comparisons were made between ten samples from chronically infected individuals before (T1 or Ss+PreT) and after (T2 or Ss+PostT) treatment. In which, samples from Neg and Ss+PostT were defined to be negative with *S. stercoralis* infection by conventional PCR.

#### Gut bacterial diversity

**Figure 1:**
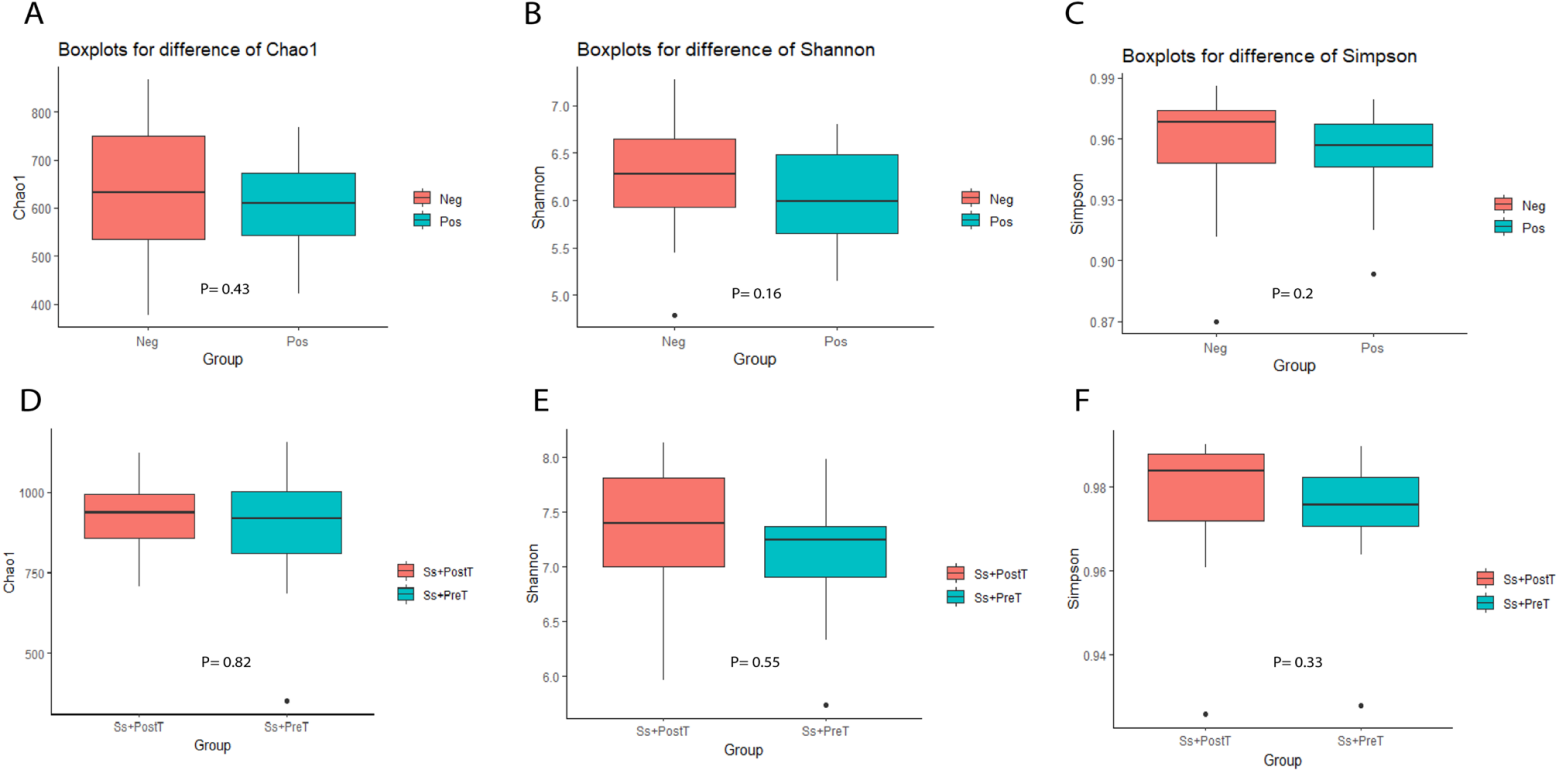
Alpha diversity of groups compared at based line between Pos and Neg (T0), and at 1 + years later between before (Ss+PreT, T1) and after (Ss+PostT, T2) presented in boxplots for Chao 1 (**A**,**D**), Shannon (**B**,**E**) and Simpson indices (**C**,**F**) with *p* values.

The intestinal microbial diversity of all fecal samples was explored using 16S-rRNA NGS data to determine whether this could be related to *S. stercoralis* infection. All alpha-diversity indices were slightly higher in Neg compared to Pos, but there were no significant differences between these groups according to Chao1, Shannon or Simpson indices at baseline (T0). Alpha diversity was compared between samples collected at T1 and T2 (Ss+PreT and Ss+PostT) but revealed no statistically significant differences between these two time points. Figure 1 depicts the alpha diversity with *p* values for all groups.

**Figure 2:**
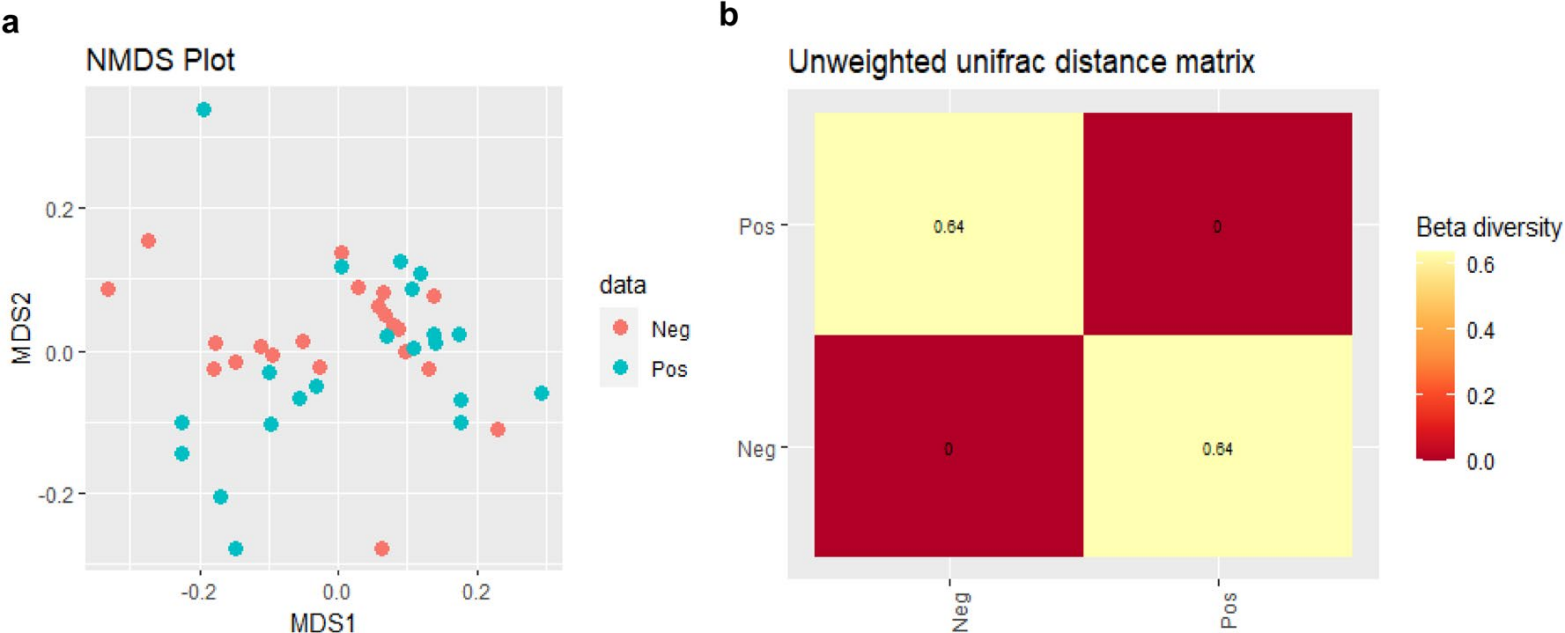
Beta diversity of samples at T0 of 16S-rRNA sequences demonstrated the distribution of samples in red dots (Neg) and green dots (Pos) in an NMDS plot (**a**). Heatmap of unweighted-Unifrac distance matrix showed a presence of some bacterial taxa only in the Pos group (index = 0.64) (**b**).

Beta diversity was evaluated using non-metric distance scaling (NMDS) analysis of the Bray–Curtis dissimilarity distance (Fig. 2a). The weighted-Unifrac distance matrix showed similar bacterial communities in participants of both groups (index = 0.11). This suggested a similar species composition between Pos and Neg samples at T0. The unweighted-Unifrac distance matrix was most efficient in detecting abundance changes in rare lineages (index = 0.64) (Fig. 2b). The result from Adonis showed no significant difference in the weighted-Unifrac distance matrix (R2 = 0.038, P = 0.106) between Pos and Neg at baseline, but there was a significant difference according to the unweighted-Unifrac distance matrix (R2 = 0.037, P = 0.028). The same analysis was also done between T1 and T2 (Ss+PreT and Ss+PostT), but there were no significant differences detected in any analyses (Supplementary Fig. S1 and Table S3).

#### Relative abundance of the most-represented microbial taxa

**Figure 3:**
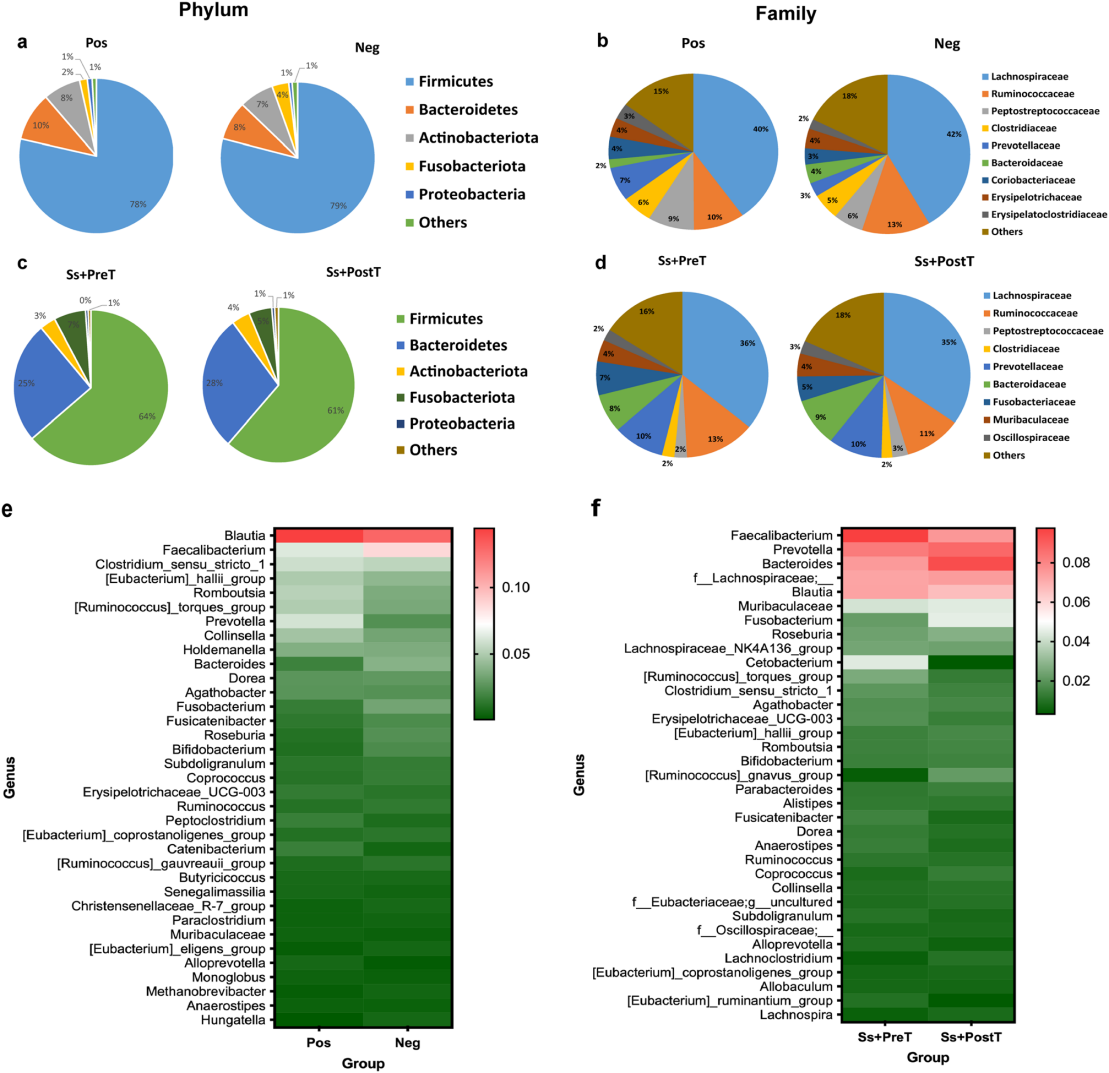
Relative abundances of the five most-represented phyla (**a**,**c**) ten most-represented families based on 16S-rRNA NGS data (**b**,**d**) in each dataset at based line between Pos and Neg (T0), and at 1 + years later between before (Ss+PreT, T1) and after (Ss+PostT, T2) treatment. Relative abundance of top 35 genera presented as heatmaps (**e**,**f**).

The five most-represented phyla and ten most-represented families (based on 16S-rRNA NGS data) are shown in Fig. 3a,c. *Firmicutes*, *Bacteroidetes*, *Actinobacteriota*, *Fusobacteriota*, and *Proteobacteria* were the five major phyla present in all groups, especially in the infected group*. Firmicutes* dominated (78.5% and 63.7% in Pos at T0 and Ss+PreT, respectively), followed by *Bacteroidetes* (10.2% and 25.2% in Pos at T0 and Ss+PreT, respectively). *Proteobacteria* had the lowest abundance among the top five phyla (1% and 0.7% in Pos and Neg, respectively). Only *Proteobacteria* were significantly enriched in individuals infected with *S. stercoralis* at baseline (Pos group) according to LEfSe analysis (Supplementary Fig. S2). At the family level (Fig. 3b,d), *Lachnospiraceae, Ruminococcaceae, Prevotellaceae, Bacteroidaceae, Clostridiaceae* and *Peptostreptococcaceae* were prominent families in the human gut microbiome. There was enrichment in the relative abundances of *Fusobacteriaceae, Muribaculaceae* and *Oscillospiraceae* at T1 and T2 (immediately before and four months after treatment), replacing *Coriobacteriaceae, Erysipelatoclostridiaceae, Erysipelotrichaceae* at baseline T0. However, none of the differences among the most-represented bacterial families reported here reached the level of statistical significance.

**Figure 4:**
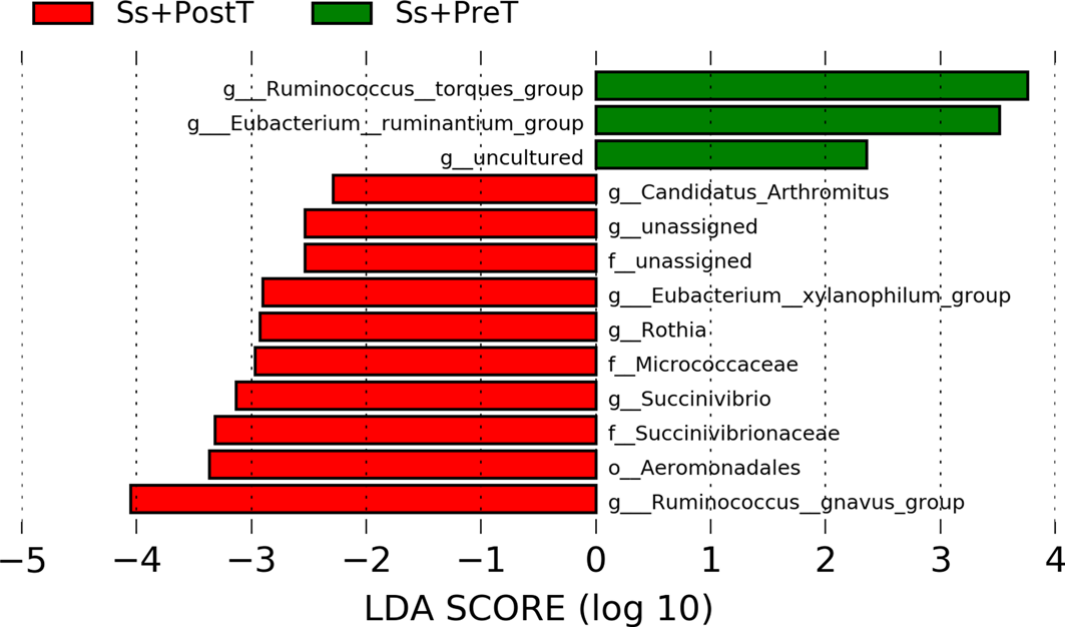
Histograms of linear discriminant analysis (LDA) effect size (LEfSe) comparison between stool microbiota at the genus level between Ss+PreT (n = 10) and Ss+PostT (n = 10). Log-level changes in LDA score are displayed on the x axis. Green and red bars taxa found in greater relative abundance in Ss+PreT and Ss+PostT, respectively.

**Figure 5:**
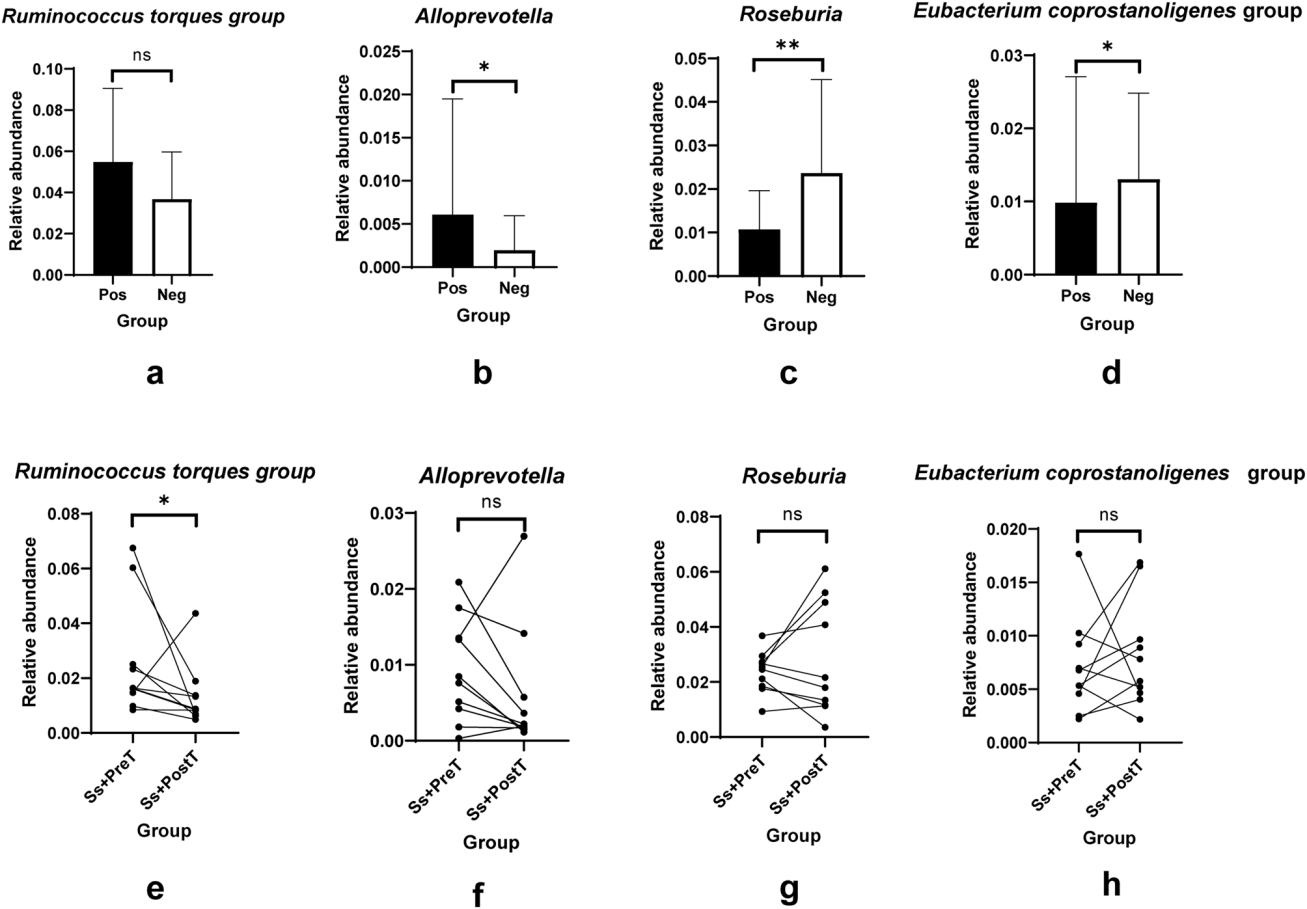
Top four genera significantly differs between uninfected groups and those with chronic infection of *S. stercoralis* based on 16S-rRNA sequences. Data at T0 are presented as box plots [(**a**,**b**,**c**,**d**) are presented for *Ruminococus torques* group, *Alloprevotella*, *Roseburia*, *Eubacterium coprostanoligenes* group, respectively] and those of the before and after treatment groups (T1 and T2) are demonstrated as lines connecting points Ss+PreT and Ss+PostT [(**e**,**f**,**g**,**h**) are presented for *Ruminococus torques* group, *Alloprevotella*, *Roseburia*, *Eubacterium coprostanoligenes* group, respectively].

The 35 most-represented genera of bacteria are shown in a heatmap (Fig. 3e,f). Five genera diminished in abundance in *S. stercoralis*-infected individuals. These were *Eubacterium coprostanoligenes* group*, Bacteroides, Coprococcus, Fusobacterium* and *Roseburia*. Five genera that increased in relative abundance in *S. stercoralis*-infected individuals were *Blautia*, the *Ruminococcus torques* group, *Clostridium sensu* *stricto* *1*, *Erysipelotrichaceae UCG-003* and *Alloprevotella*. Differences at the genus level were also analyzed using LefSe (Fig. 4 and Supplementary Fig. S2). This also revealed that abundance of the *R. torques* group and of *Alloprevotella* was significantly boosted in the presence of *S. stercoralis* (*R. torques* group (Fig. 5a, *p* > 0.05; Fig. 5e, *p* = 0.048) and *Alloprevotella* (Fig. 5b, *p* = 0.04; Fig. 5f, *p* > 0.05)*.* In contrast, *Roseburia* and the *E. coprostanoligenes* group exhibited significantly reduced abundance during helminth infection (*Roseburia* (Fig. 5c, *p* = 0.0057; Fig. 5g, *p* > 0.05) and *E. coprostanoligenes* (Fig. 5d, *p* = 0.0146; Fig. 5h, *p* > 0.05). Among the top four most-represented genera, *R. torques* showed the greatest changes in abundance between infected and uninfected individuals: abundance of this genus was reduced in nine out of ten samples after treatment.

### Metaproteomic results

#### Fecal proteins derived from members of the ten most-abundant bacterial families before and after treatment of chronic strongyloidiasis

**Figure 6:**
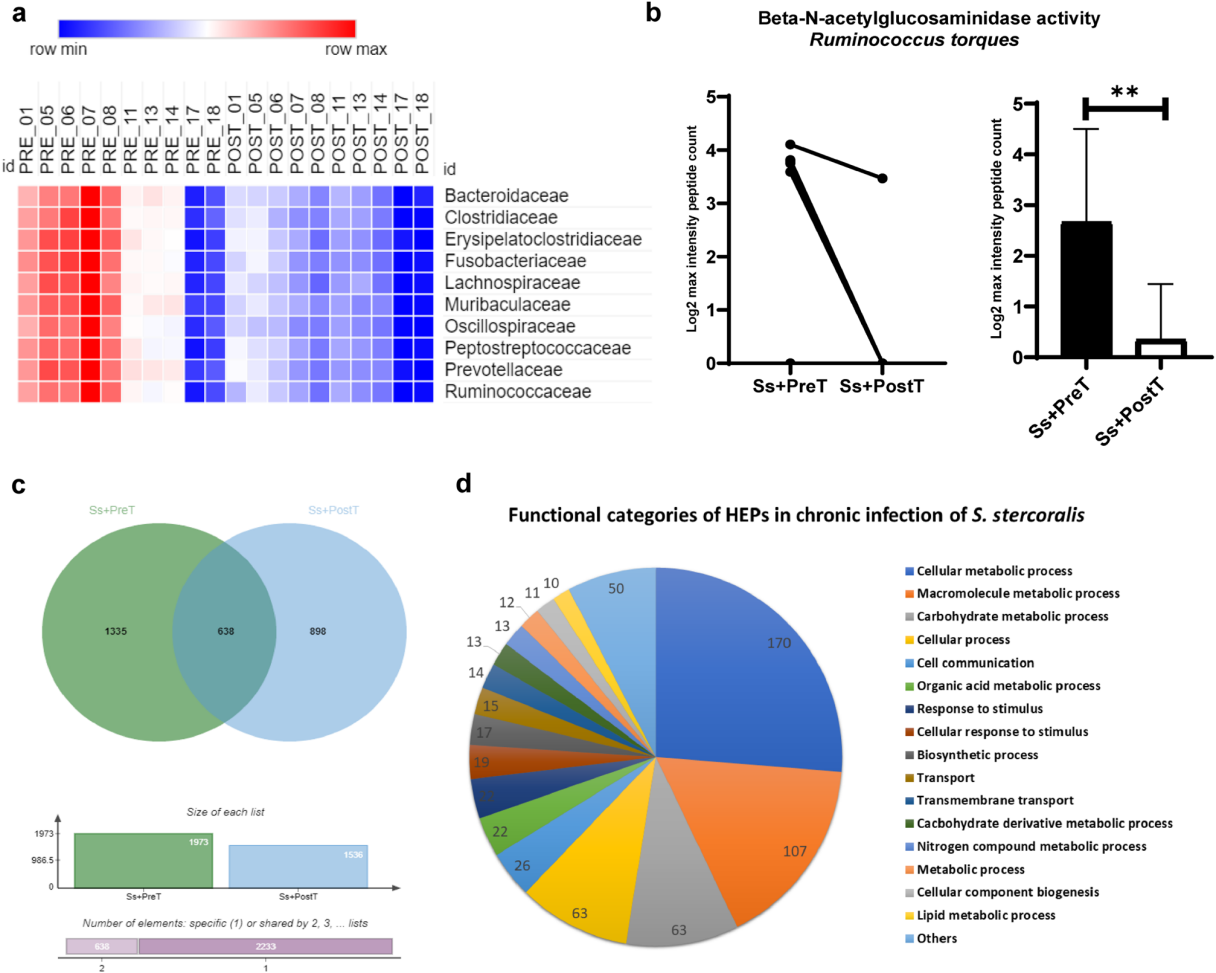
Metaproteomic analysis result. (**a**) Heatmap of abundance of fecal proteins derived from the top 10 bacterial families for ten individuals both before (Ss+PreT (T1)) and after ivermectin treatment (Ss+PostT (T2)). (**b**) Relative levels of the beta-N-acetylglucosaminidase enzyme of *Ruminococcus torques* expressed in Ss+PreT and Ss+PostT groups by log2 max intensity peptide count. Data are presented as line graphs pre- and post-treatment of individual samples (7/10 samples contained this protein) and as a bar chart. Each column shows data from a single individual. (**c**) Jvenn diagram showing numbers of highly expressed proteins (HEPs) in Ss+PreT and Ss+PostT groups and (**d**) functional categories of HEPs in Ss+PreT in a pie chart.

To compare levels of protein expression in the ten most-abundant bacterial families between before (Ss+PreT) and after treatment (Ss+PostT) of chronic strongyloidiasis, the maximum intensity of protein in each sample was counted to determine total number of proteins representation at the family level. A total of 52,798 proteins, including uncharacterized proteins, derived from the top 10 families were assessed (Table 1). The relative decrease in protein numbers in the top 10 bacterial families after treatment is shown in the heatmap (Fig. 6a). However, there were no significant differences among all families after applying false discovery rate, FDR test (FDR = 1%) (Table 1).

**Table 1:** Summary of number of fecal proteins ascribed to the ten most abundant bacterial families. Adjusted *p* value < 0.01 is considered as indicating a significant difference (n = sample size of each group).

Family	Ss+PreT (n = 10)	Ss+PostT (n = 10)	Adjusted *p* value
*Bacteroidaceae*	2724.6 ± 419.4	2291 ± 211.3	0.012
*Clostridiaceae*	2987.2 ± 458.6	2468.4 ± 250.6	0.012
*Erysipelatoclostridiaceae*	2890.4 ± 449.9	2435.7 ± 228.1	0.012
*Fusobacteriaceae*	2915.7 ± 454.1	2432.5 ± 226.8	0.012
*Lachnospiraceae*	2809.5 ± 442.3	2335.1 ± 224.2	0.012
*Muribaculaceae*	2659.8 ± 407	2198.3 ± 215.5	0.012
*Oscillospiraceae*	3030.1 ± 470.6	2498.6 ± 237.1	0.012
*Peptostreptococcaceae*	2907.1 ± 467.7	2459 ± 243.5	0.015
*Prevotellaceae*	3005.9 ± 460.7	2462.9 ± 257.2	0.012
*Ruminococcaceae*	2653.7 ± 422.8	2214.3 ± 213.3	0.012

#### Differentially expressed proteins compared before and after treatment of chronic strongyloidiasis

Differentially expressed proteins (DEPs) analysis with FDR testing (p-adjusted < 0.05) revealed no significant differences among the top ten families. *Ruminococcus torques* was conspicuously the bacterium with the most-altered abundance during *S. stercoralis* infection according to 16S-rRNA NGS data. This taxon is well-known for its mucin-degrading properties. All metaproteomic data of *Ruminococcus* spp*.* were therefore examined for the presence of proteins related to mucus glycoproteins without FDR testing. We found one protein, named F5/8 type C domain-containing protein of the *R. torques* group, which was classified by GO function as having beta-N-acetylglucosaminidase activity [GO:0016231] (Supplementary file Excel 1). This protein was significantly reduced after treatment by ivermectin (seven out of ten samples contained this protein (see Fig. 6b)).

#### Functional categories of highly expressed proteins in chronic *S. stercoralis* infection

Because DEPs analysis with FDR testing (p-adjusted < 0.05) revealed no significant differences among the top ten families, we focused on highly expressed proteins (HEPs) in chronic *S. stercoralis* infection. Only proteins present in at least 50% of samples in each group were selected to be analyzed by Venn diagrams (Fig. 6c). A total of 1335 proteins were highly represented in Ss+PreT samples across all 10 bacterial families (Supplementary Excel file 2). Among these, 647 HEPs were assigned to biological processes according to Uniprot and subcategorized into different functions (see Fig. 6d and Supplementary Excel file 3). Proteins associated with carbohydrate metabolism were markedly enriched in samples from people with *S. stercoralis* infection (Fig. 6d) and most of these were produced by members of the *Bacteroidaceae*. Indeed, the most-represented category of HEPs produced by this family of bacteria was associated with carbohydrate metabolism (Supplementary file Excel 3). Thus, proteins produced by this family were selected for further analysis using STITCH, focusing on carbohydrate metabolism.

#### STITCH analysis network of HEPs of *Bacteroidaceae* during chronic *S. stercoralis* infection

**Figure 7:**
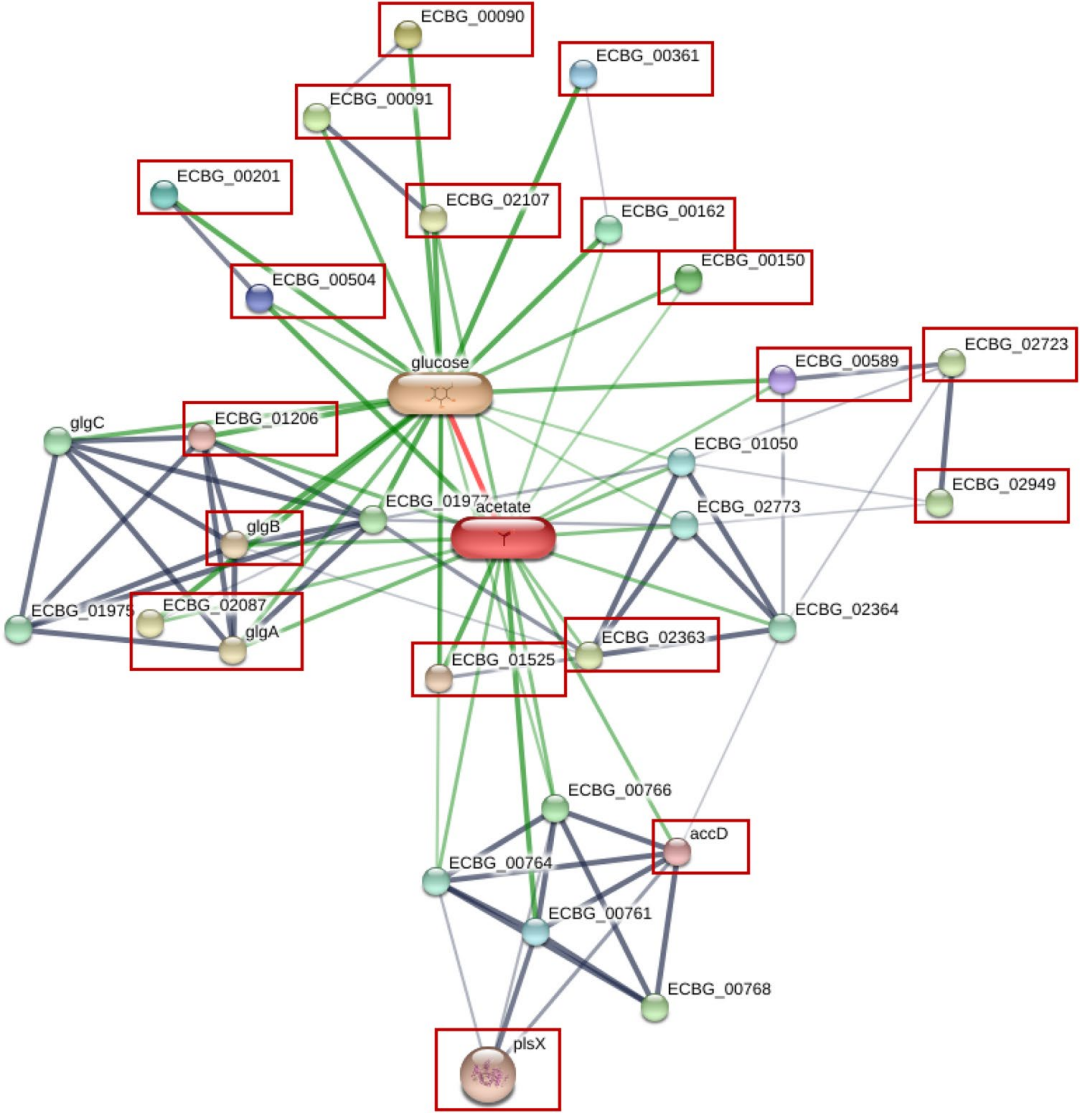
STITCH analysis network of HEPs and SCFA molecules with *S. stercoralis* infection. Know/putative proteins among the highly expressed proteins (HEPs) of the Ss+PreT group of samples are marked in red boxes.

The interesting proteins from *Bacteroidaceae* associated with carbohydrate metabolism were subjected to STITCH analysis to identify the enrichment pathway in *S. stercoralis* infection. The results demonstrated that carbohydrate metabolism from this family of bacteria is mainly involved in cellulose metabolism (Supplementary Fig. S3 and Table S4). Humans cannot digest cellulose, but intestinal bacteria do so to produce short-chain fatty acids (SCFAs) such as acetate. HEPs assigned to carbohydrate and fatty-acid metabolism were highly represented in Ss+PreT samples. STITCH analysis revealed interactions between known or putative proteins of Ss+PreT and acetate production (*Enterococcus casseliflavus* was used as a template) (see Fig. 7 and Supplementary file Excel 4).

## Discussion

In this study, we investigated the impact of *S. stercoralis* infection on gut microbiota in participants considered otherwise healthy in a community at Nam Phong and Ubonrat Districts, Khon Kaen Province, Northeast Thailand. Longitudinal evaluation during chronic infection with *S. stercoralis* and following deworming treatment allowed us to demonstrate the real effect of *S. stercoralis* on the host’s gut microbiome. This is also the first study to explore metaproteomic-based analysis in research on the human gut microbiome after deworming. Based on this, we discovered that helminth infection alters not only the composition of the gut microbiome but also the metabolic function of that community. In our study, ivermectin was given as a single dose, so the alterations in our study, especially four months posttreatment, were considered a consequence of worm clearance rather than of the anthelmintic treatment itself^11^.

Our research focused on otherwise healthy individuals with chronic mono-infection with *S. stercoralis* in endemic areas and did not find any significant alteration in alpha diversity. Intestinal helminths inhabiting the large intestine, have a stronger effect on gut microbiota compared to those in the small intestine^11^. In our study, chronic *S. stercoralis* infection, persisting in the small intestine with a low worm burden (no larvae were found by FECT at T1) might not significantly alter measures of gastrointestinal microbial diversity. For beta diversity, we found only dissimilarity of taxonomic content between Pos and Neg groups at baseline (T0) according to the unweighted-Unifrac distance matrix, demonstrating the presence of some specific bacterial taxa associated with parasite infection. Unweighted-UniFrac is more sensitive to differences in low-abundance features. Other reports from Thailand concerning soil-transmitted helminths (*Ascaris* sp, hookworm and *Trichiuris trichiura*) in children showed similar results with no difference between infected and uninfected groups in bacterial alpha-diversity but did observe differences in beta-diversity^19^. In previous research we conducted in Don Chang District, Khon Kaen Province, alpha diversity was found to be greater in infected individuals. Another recent study in Thailand showed that bacterial diversity differed between subjects from different schools, possibly due to inter-school variation in diet^19^. Thus, different districts and time-points of sample collection could affect gut diversity in the same general area. The impact of helminths on the gut microbiota likely varies due to the large range of environmental variables (diet, sanitation, species of parasite, worm burden, etc.) and the batch effect produced by sample numbers and collection^11^.

Analysis of the 35 most-represented bacterial genera demonstrated many shifts in relative abundance of these between Pos versus Neg at baseline and 1 + years later between the Ss+PreT and Ss+PostT groups. Among these changes, the *Ruminococcus torques* group considerably increased in *S. stercoralis* infection. This taxon is enriched in various clinical situations such as visceral obesity, hemodialysis, and irritable bowel syndrome (IBS) or children’s autism-spectrum disorders^20–23^. In contrast, depletion of *Ruminococcus* during *S. stercoralis* infection has been reported in another study in a non-endemic area^15^. Our previous study with *S. stercoralis* infection in Donchang did not find a similar alteration in the *R. torques* group^14^. Different districts, the time of sample collection, and varying levels of infection could all account for the variation in the findings. Furthermore, in our previous study, samples from 26 individuals had been pooled whereas we analyzed samples individually in this study. In addition, we also evaluated samples from the same ten individuals at T0 and T1 to investigate alterations in bacterial genera, particularly the *R. torques* group, during chronic infection. However, in general, there was considerable difference in bacterial genera present at T0 and T1 (data not shown). Factors other than infection could have been affecting *R. torques* populations during the long and enforced gap between sample collections during the COVID-19 pandemic^24^. Thus, in this study, we decided to analyze our material as two separate sets of 16S-rRNA, one at baseline (T0), comparing Pos and Neg, and the other at T1 plus T2, comparing chronically infected individuals before and after worm clearance.

The *Ruminococcus torques* group became enriched during chronic *S. stercoralis* infection, more so than any other taxon according to the 16S-rRNA NGS data, and showed a significant reduction after treatment in nine out of ten samples (Fig. 4). Members of this group are known to produce substances that strongly degrade gastrointestinal mucin and play a role in the pathophysiology of Crohn’s disease^25–27^. Mucin is a major structural and functional component present in the mucus at concentration of 1–5%^28^. Large amounts of mucus in stool are associated with diarrhea in conditions such as Crohn’s disease or IBS^29^. Similarly, infections with *S. stercoralis* or other helminths also increase mucus production with frequent diarrhea. The mucosal integrity of the entire gastrointestinal (GI) tract is vital for maintaining health. Given the known mucin-degrading ability of the *R. torques* group, we investigated in more detail the metaproteomic data from this taxon. One protein linked to beta-N-acetylglucosaminidase from *R. torques* CAG 126:1 was found to decrease significantly after treatment (Fig. 6b). Beta-N-acetylglucosaminidase is putatively involved in mucin degradation^28^. Mucus provides a crucial innate defense against invading gastrointestinal parasitic nematodes^30,31^, so we hypothesize that an increase of *R. torques* during chronic infection might aid prolonged worm survival in the host by degradation of mucus. An increase of the *R. torques* group may facilitate the establishment of new parthenogenetic females because of reduced mucus-mediated expulsion. While the same group of mucus-associated bacteria^28^ has been found in Crohn’s disease, there is a negative correlation between *A. muciniphilia* and *R. torques*^25,32^. Unlike *Akkermansia*, a well-known beneficial bacterial taxon^33,34^, an increase of *R. torques* has been highlighted in other diseases^25–29^. Further studies should be done to confirm the important roles of *R. torques* during in chronic *S. stercoralis* infection.

In this study, we also analyzed metaproteomic data before and after treatment of chronic *S. stercoralis* infection. There remain many knowledge gaps in our ability to evaluate effects of worm clearance on gastrointestinal microbes. Most studies have used 16S-rRNA sequences, which is unable to provide information about the functional roles of the bacterial community. Metaproteomics serves as a novel useful tool to investigate treatment effects on human gut microbes and has been applied here for the first time. Because no differentially expressed proteins (DEPs) were found by the TM4 suite of software, we focused on 1335 highly expressed bacterial proteins (HEPs) that were present in participants prior to treatment. We found that many of these were associated with carbohydrate metabolic function (Fig. 6d). This was especially the case for members of the *Bacteroidaceae*. A previous study using metagenomic profiles also predicted an enrichment of carbohydrate metabolism during infection with *S. stercoralis*^15^. HEPs present in helminth infection and originating from members of the *Bacteroidaceae* were recruited for STITCH analysis linked to glucose metabolism derived from cellulose digestion. Cellulose is a major building block of plant cell walls, consisting of molecules linked together into solid fibers. For humans, cellulose is indigestible, so gut bacterial enzymes are required to break down this material. One of the final products from cellulose digestion of bacteria is short-chain fatty acids (SCFAs). Bacterial interactions, particularly cross-feeding, which is the use of substrates or metabolites generated by other bacteria, are important in determining the final amounts of SCFAs in the colon because bacterial taxa do not act in isolation^35^. Many of the HEPs related to carbohydrate metabolism from infected individuals identified in this study were highly represented among enzymes related to acetate according to our STITCH network. When compared to other SCFAs, acetate often reaches the highest levels in the colon and can be produced by many gut microbial species^35^. In contrast, analysis of fecal samples found higher levels of acetate in non-infected subjects than in *S. stercoralis*-infected subjects in non-endemic areas^13^. Relevantly, decreased serum acetate level was also observed in strongyloidiasis in an endemic area^14^. Contradictory findings of acetate level between serum and fecal samples may be due to a variety of other factors related to SCFA production, not only *S. stercoralis* infection^16,35–37^. In addition, *Bacteroidaceae* also harbors several mucin-degrading groups and several of the glycosyl hydrolases identified in Supplementary Table S4 degrade dietary components as well as mucin glycans^38,39^. This result supports our hypothesis, mentioned earlier, that altered mucin profiles may support parasite persistence.

In conclusion, chronic *S. stercoralis* infection in infected participants at Nam Phong and Ubonrat districts did not lead to altered gut bacterial diversity. When compared to other taxa, the *R. torques* group has the largest alteration in abundance during chronic *S. stercoralis* infection. Interestingly, this, together with an increase in mucin degrader enzyme produced by this taxon, may facilitate prolonged worm persistence. Furthermore, anti-helminthic treatment and worm clearance with ivermectin depleted carbohydrate metabolism, with *Bacteroidaceae* being the primary culprits. Acetate production was likely to be increased during *S. stercoralis* infection based on STITCH analysis networks. Taken together, our results indicate that chronic *S. stercoralis* infection not only changes the human gut microbiota but also alters metabolic homeostasis.

## Materials and methods

### Human ethics statement

This study was approved by the Human Ethical Review Committee of Khon Kaen University, Thailand (HE641434) following the principles of the Declaration of Helsinki. Before stool samples were collected, participants were required to complete and sign written informed-consent forms.

### Participants and samples

All samples came from residents of Nam Phong and Ubonrat Districts, Khon Kaen Province, Northeast Thailand, under the Chronic Kidney Disease Northeastern Thailand (CKDNET) program. Intestinal parasitic infections were diagnosed from stool samples using the modified agar plate culture (mAPC) method and the formalin-ether concentration technique (FECT) as previously reported^40^. The inclusion criteria for the current investigation were infection with *S. stercoralis* alone (no additional intestinal parasites) and no recent usage of antibiotics or ivermectin as determined by questionnaires. At the times of sample collection, none of the patients was using medications such as corticosteroids, immunomodulators, or biological agents and were apparently otherwise healthy based on the questionnaires. None of the patients had underlying conditions such as diabetes and hypertension. The summary of biochemical tests of participants and sample details at each time-point are presented in Supplementary Tables S1 and S2, respectively.

The baseline samples we used were leftover fecal specimens from 42 individuals collected as part of an earlier study, in which a single stool sample was screened from each of 704 subjects^40^. From among these 704 subjects, we selected 21 positive cases and 21 matched negative cases. Of these specimens (baseline, or time-point T0), the samples were matched between groups by gender, age and were in the normal range for all biochemical tests done. Infected individuals at T0 could not be treated following this initial diagnosis because of the extended lockdowns imposed by the Thai Government as a result of the COVID19 pandemic. A stool sample was collected from each of the positive subjects 12 months later (T1), and examined using both diagnostic methods. Ten of these were collected and found to still be infected with *S. stercoralis* with low intensity of infection (no larvae were detected by FECT but larvae were still found using the agar plate technique). No other species of parasite was found. Since no subjects had previously received any ivermectin treatment, we regarded them as suffering chronic infection. Those fecal samples were coded as “Ss+PreT” and their donors were treated with ivermectin (200 μg/kg body weight). Around four months later (time-point T2), fecal samples were again obtained from these individuals and coded as “Ss+PostT”. Fecal examination showed all ten to be free of *S. stercoralis* infection. After being collected in the field, fecal samples (Ss+PreT and Ss+PostT) were quickly transferred to ice and frozen at -80 °C until analysis.

### Polymerase chain reaction (PCR) confirmation of presence or absence of *S. stercoralis* infection

Conventional PCR was used to confirm the findings of fecal examination, including for samples that were fecal-negative. Primers were those used in a previous study to amplify a 230-bp fragment from a dispersed repetitive sequence of *S. stercoralis*, GenBank accession no. M84229.1^41^. The forward primer was 5′-CCGGACACTATAAGGATTGA-3′, and the reverse was 5′-ACAGACCTGTTATCGCTCTC-3′. The amplification profile was initial denaturation at 94 °C for 5 min; and 35 cycles at 94 °C for 40 s, 52.8 °C for 30 s, 72 °C for 2 min; and a final extension at 72 °C for 10 min. To confirm amplification and amplicon size, the PCR products were resolved on a 1.5% agarose gel stained with a non-toxic dye.

### Fecal DNA extraction and Illumina sequencing

DNA was extracted from all 62 stool samples using QIAamp PowerFecal Pro DNA Kit (QIAGEN, Hilden, Germany) according to the manufacturer’s instructions. Samples were checked by agarose gel electrophoresis and an Agilent5400 fragment-analyzer system before library construction. The V3-V4 region of the prokaryotic 16S-rRNA gene was PCR-amplified and sequenced on an Illumina platform. In summary, targeted regions were PCR-amplified using specific primers connected to barcodes^42^. PCR products of the proper size were selected by agarose gel electrophoresis and equal quantities from each sample were pooled and Illumina adapters ligated after being end-repaired. The library was examined using a Qubit fluorometer, real-time PCR, and Bioanalyzer to check size distribution and for quantification. Libraries were sequenced on a paired-end Illumina platform to generate 250-bp paired-end raw reads.

### Sample preparation for shotgun proteomics

A volume of 500 µl of 0.5% sodium dodecyl sulphate (SDS) was added to 100 mg feces, mixed well by pipetting, vortexed and centrifuged at 10,000*g* for 15 min. The supernatant was transferred to a new tube, mixed well with 2 volumes of cold acetone, and incubated overnight at − 20 °C. The mixture was centrifuged at 10,000*g* for 15 min to remove the supernatant; the protein pellet, after precipitation, was then dried and stored at − 80 °C prior to analysis. The protein concentration of each sample was then determined by the Lowry assay using bovine serum albumin as a standard protein^43^. Disulfide bonds were broken down in the presence of 10 mM dithiothreitol in 10 mM ammonium bicarbonate, and the reformation of disulfide bonds in the proteins was prevented by alkylation with 30 mM iodoacetamide in 10 mM ammonium bicarbonate. Sequencing-grade porcine trypsin was used to digest the protein samples for 16 h at 37 °C. For analysis by nano-liquid chromatography tandem mass spectrometry (nano LC–MS/MS), the tryptic peptides were dried with a speed vacuum concentrator and resuspended in 0.1% formic acid.

### Liquid chromatography-tandem mass spectrometry (LC/MS–MS)

Thermo Scientific's Ultimate3000 Nano/Capillary LC System (Thermo Scientific, UK) was used to analyze the tryptic peptide samples. It was connected to a hybrid quadrupole Q-ToF impact II (Bruker Daltonics Ltd; Hamburg, Germany) with a Nano-captive spray ion source. Peptide digests were packed with Acclaim PepMap RSLC C18, 2 µm, 100 Å, and nanoViper after being enriched on a µ-Precolumn 300 m i.d. X 5 mm C18 PepMap 100, 5 µm, 100 Å (Thermo Scientific, UK) (Thermo Scientific, UK). A thermostated column oven with a 60 °C setting was used to surround the C18 column. On the analytical column, solvents A and B. containing 0.1% formic acid in water and 0.1% formic acid in 80% acetonitrile, respectively, were provided. The peptides were eluted using a gradient of 5–55% solvent B for 30 min at a constant flow rate of 0.30 µl/min. The CaptiveSpray was used for 1.6 kV electrospray ionization. About 50 l/h of nitrogen was employed as a drying gas. When nitrogen gas served as the collision gas, collision-induced-dissociation (CID) product ion mass spectra were obtained. Positive-ion mode mass spectra (MS) and MS/MS spectra were obtained at 2 Hz over the (m/z) 150–2200 range. Based on the m/z value, the collision energy was modified to 10 eV. Each sample was examined in triplicate using LC–MS.

### Bioinformatics and data analysis

#### 16S-rRNA NGS bioinformatic analysis

Raw data were further processed using Quantitative Insights into Microbial Ecology 2 (QIIME2) version qiime2-2021.11, a software package that performs microbial community analysis and taxonomic classification of microbial genomes^44^. Demultiplexing was conducted to remove primer sequences and paired-end reads were assigned to individual samples based on their unique barcode. Quality filtering on the raw reads was performed under specific filtering conditions (Q Score > 33) to obtain the high-quality clean reads. Denoised paired-end reads, after quality filtering by DADA2, were clustered into operational taxonomic units (OTUs) based on a criterion of 97% similarity. Naïve Bayes Classifiers of the Silva full-length database were applied to classify taxonomy. For diversity analysis, samples were normalized so that all could be compared. Alpha diversity of OTU libraries was described using the Chao1, Simpson and Shannon indices using Kruskal–Wallis tests. Non-metric distance scaling (NMDS) analysis of the Bray–Curtis dissimilarity distance of samples among groups. The distance matrices were constructed using the unweighted and weighted UniFrac algorithms in QIIME2 from the whole-community phylogenetic tree. Adonis in QIIME2 was applied to calculate statistically significant differences for beta diversity among groups. Gut diversity was visualized using R studio version 2022.7.2.576 (http://www.rstudio.com/). The most abundant bacteria phyla and families were entered into Excel spreadsheets. The heatmap showing the top 35 bacterial genera was constructed using GraphPad Prism version 8.4 (www.graphpad.com).

A linear discriminant analysis (LDA) effect size (LEfSe) score criterion of > 2.0 was used to identify features that were significantly different in abundance between the groups in order to identify possible biomarker OTUs^45^. All significance thresholds were set at a two-sided *p* value of 0.05. Data of population characteristics and bacterial compositions (*p* value) were analyzed and visualize using GraphPad Prism version 8.4 and Excel.

### Metaproteomic analysis of samples before and after treatment

Proteins in individual samples were bioinformatically quantified by MaxQuant 2.1.0.0 using the Andromeda search engine to correlate MS/MS spectra to proteins produced by the ten most abundant bacterial families (*Lachnospiraceae, Ruminococcaceae, Prevotellaceae, Bacteroidaceae, Fusobacteriaceae, Muribaculaceae, Peptostreptococcaceae, Oscillospiraceae, Clostridiaceae, Erysipelatoclostridiaceae*) and one particular genus (*Ruminococcus*)*.* The Uniprot database, which was accessed on 15 June 2022, was used for this. Label-free quantitation with MaxQuant’s standard settings was performed using a maximum of two missed cleavages, mass tolerance of 0.6 Dalton for the main search, trypsin as the digesting enzyme, carbamidomethylation of cysteine as a fixed modification, and oxidation of methionine and acetylation of the protein N-terminus as variable modifications. Peptides with a minimum of seven amino acids and at least one unique peptide were considered for protein identification and used for further data analysis. The protein FDR was set at 1%. The maximum number of modifications per peptide was set to 5. Peptides with maximum intensity from three injections were detected as spectral data of the total proteins expressed from each taxon.

Maximum peptide intensities were log2 transformed in Microsoft Excel, providing the protein expression levels for quantification of protein numbers and analysis of differentially expressed proteins (DEPs) in the ten most abundant bacterial families. Multiple t-tests using the two-stage linear step-up procedure of Benjamini, Krieger and Yekutieli^46^ (false discovery rate, FDR = 1%), carried out in GraphPad Prism, were applied to analysis of number of proteins of top 10 bacterial families of the 2 groups at T1 and T2. Numbers of proteins from each family were visualized in a heatmap using Morpheus from the Broad Institute (https://software.broadinstitute.org/morpheus). Log2 of max protein intensity from each sample was then subjected to statistical analysis to determine DEPs by using the MultiExperiment Viewer (MeV) in the TM4 suite software (adjusted *p* value < 0.05) in the 10 selected bacterial families^47^. The Wilcoxon rank-sum test with multiple-testing FDR correction was performed. The same analysis was applied for the genus *Ruminococcus* alone without applying a FDR correction. Only proteins found in at least 50% of all samples in each group were further selected for analysis of highly expressed proteins (HEPs) using the Jvenn viewer^48^. Proteins found only in Ss+ PreT were considered as enriched proteins or HEPs. Those HEPs, present during chronic *S. stercoralis* infection, were classified further into different metabolic categories through GO biological function categories from Uniprot. The STITCH database version 5 was used to forecast functional interaction networks between identified proteins and small molecules and to generate figures (http://stitch.embl.de/)^49^.

## Supplementary Information

Supplementary Information 1.

Supplementary Information 2.

## Data Availability

The raw sequencing data have been deposited on the Sequencing Reads Archive (SRA), and SRA accession numbers are PRJNA905167 (https://www.ncbi.nlm.nih.gov/sra/PRJNA905167). The MS proteomics data in this paper have been deposited in the ProteomeXchange Consortium (http://www.proteomexchange.org/) via the jPOSTrepo partner (https://repository.jpostdb.org/preview/4809657056368e82009baa) with access key is 6992.
