# Supplementary material for: Chronic Strongyloides stercoralis infection increases presence of the Ruminococcus torques group in the gut and alters the microbial proteome
Source: Sci Rep. 2023 Mar 14;13:4216. doi: 10.1038/s41598-023-31118-5 (PMC10012286; doi:10.1038/s41598-023-31118-5)
Supplement: Supplementary file 1 — Supplementary Information 1. [file 41598_2023_31118_MOESM1_ESM.docx]

**Chronic *Strongyloides stercoralis* infection increases presence of the *Ruminococcus torques* group in the gut and alters the microbial proteome**

**Supplementary information**

Na T. D. Tran^1,7^, Apisit Chaidee^1,7^, Achirawit Surapinit^1^, Manachai Yingklang^2^, Sitiruk Roytrakul^3^, Sawanya Charoenlappanit^3^, Porntip Pinlaor^4,7^, Nuttanan Hongsrichan^1,7^ Sirirat Anutrakulchai^5,7^, Ubon Cha’on^6,7^, Somchai Pinlaor^1,7*^

^1^Department of Parasitology, Faculty of Medicine, Khon Kaen University, Khon Kaen, Thailand

^2^Faculty of Public Health, Burapha University, Chonburi, Thailand

^3^Functional Proteomics Technology Laboratory, National Center for Genetic Engineering and Biotechnology, National Science and Technology Development Agency, Pathum Thani, Thailand

^4^Centre for Research and Development of Medical Diagnostic Laboratories, Faculty of Associated Medical Sciences, Khon Kaen University, Khon Kaen, Thailand

^5^Department of Medicine, Faculty of Medicine, Khon Kaen University, Khon Kaen, Thailand

^6^Department of Biochemistry, Faculty of Medicine, Khon Kaen University, Khon Kaen, Thailand

^7^Chronic Kidney Disease Prevention in Northeastern Thailand, Khon Kaen University, Khon Kaen , Thailand

*Corresponding author. Somchai Pinlaor (E-mail: psomec@kku.ac.th)

**Supplementary Table S1**. Characteristics of participants in study at the baseline (T0). Pos and Neg indicate the groups of individuals positive and negative, respectively, for *S. stercoralis* infection; n = sample size in each group. An adjusted p-value < 0.01 is considered as indicating a significant difference. Data are presented as mean ± standard deviation of the mean. Abbreviations: BMI, body max index; ALT, alanine aminotransferase; LDL, low-density lipoprotein; MCV, mean corpuscular volume; MCH, mean corpuscular hemoglobin; MCHC, mean corpuscular hemoglobin concentration.

| Parameters | Pos (n=21) | Neg (n=21) | Adjusted p-value |
| --- | --- | --- | --- |
| Age | 60.7±8.9 | 61.1±8.6 | >0.99 |
| BMI | 22.0±3.2 | 22.2±3.4 | >0.99 |
| ALT (U/L) | 22.1±10.4 | 17.7±9.3 | 0.97 |
| Serum creatinine (mg/dL) | 0.9±0.2 | 0.9±0.1 | >0.99 |
| eGFR (mL/min/1.73m^2^) | 86.3±12.0 | 85.9±11.9 | >0.99 |
| Glucose (mg/dL) | 89.0±6.7 | 89.7±6.5 | >0.99 |
| LDL-cholesterol (mg/dL) | 113.8±39.5 | 124.4±29.2 | >0.99 |
| Uric acid (mg/dL) | 5.6±1.2 | 5.2±0.9 | >0.99 |
| Red blood cell (*10^12^ /L) | 4.7±0.6 | 4.7±0.4 | >0.99 |
| Hemoglobin (g/dL) | 13.2±1.4 | 13.3±1.3 | >0.99 |
| Hematocrit (%) | 40.2±4.4 | 40.8±3.4 | >0.99 |
| White blood cell (*10^9^ /L) | 6.7±2.4 | 6.5±1.4 | >0.99 |
| MCV (fL) | 86.4±9.1 | 86.7±8.7 | >0.99 |
| MCHC (g/dL) | 32.9±0.8 | 32.5±1.2 | >0.99 |
| PLT % | 277.7±54.4 | 282.4±66.0 | >0.99 |
| NEUT % | 49.9±10.1 | 56.9±7.0 | 0.24 |
| LYMPH % | 33.0±9.4 | 32.8±6.1 | >0.99 |
| MONO % | 6.6±2.5 | 5.9±1.3 | >0.99 |
| EO % | **9.7±4.9** | **3.8±2.0** | **<0.01** |
| BASO % | 0.7±0.4 | 0.6±0.3 | >0.99 |

**Supplementary Table S2.** Details of individual samples/donors used in the study. 2 sets of **16S rRNA at T0** (Total 42 samples) and 10 samples at T1 and T2 (Total 20 samples).

1 year

| **Sample ID** | T0 | T1 (Pretreatment) | T2 (Posttreatment)  4 months |
| --- | --- | --- | --- |
| **N01** | x |  |  |
| **N02** | x |  |  |
| **N03** | x |  |  |
| **N04** | x |  |  |
| **N05** | x |  |  |
| **N06** | x |  |  |
| **N07** | x |  |  |
| **N08** | x |  |  |
| **N09** | x |  |  |
| **N10** | x |  |  |
| **N11** | x |  |  |
| **N12** | x |  |  |
| **N13** | x |  |  |
| **N14** | x |  |  |
| **N15** | x |  |  |
| **N16** | x |  |  |
| **N17** | x |  |  |
| **N18** | x |  |  |
| **N19** | x |  |  |
| **N20** | x |  |  |
| **N21** | x |  |  |
| **P01** | x | x | x |
| **P02** | x |  |  |
| **P03** | x |  |  |
| **P04** | x |  |  |
| **P05** | x | x | x |
| **P06** | x | x | x |
| **P07** | x | x | x |
| **P08** | x | x | x |
| **P09** | x |  |  |
| **P10** | x |  |  |
| **P11** | x | x | x |
| **P12** | x |  |  |
| **P13** | x | x | x |
| **P14** | x | x | x |
| **P15** | x |  |  |
| **P16** | x |  |  |
| **P17** | x | x | x |
| **P18** | x | x | x |
| **P19** | x |  |  |
| **P20** | x |  |  |
| **P21** | x |  |  |


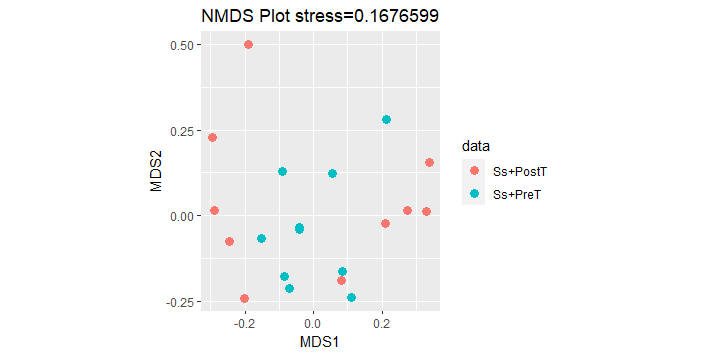


**Supplementary Figure S1**. Beta diversity of T1 versus T2 samples presented as an NMDS Plot.

**Supplementary Table S3**. Adonis analysis of Weighted and Unweighted-Unifrac distance matrices

| Pos and Neg Weighted-Unifrac | | | |  |  |  |
| --- | --- | --- | --- | --- | --- | --- |
|  | **Df** | **SumsOfSqs** | **MeanSqs** | **F.Model** | **R2** | **Pr(>F)** |
| **APC** | 1 | 0.058217 | 0.058217 | 1.578905 | 0.037974 | 0.106 |
| **Residuals** | 40 | 1.474875 | 0.036872 | NaN | 0.962026 | NaN |
| **Total** | 41 | 1.533092 | NaN | NaN | 1 | NaN |
|  |  |  |  |  |  |  |
|  |  |  |  |  |  |  |
| Pos and Neg UnWeighted-Unifrac | | | |  |  |  |
|  | **Df** | **SumsOfSqs** | **MeanSqs** | **F.Model** | **R2** | **Pr(>F)** |
| **APC** | 1 | 0.214501 | 0.214501 | 1.544159 | 0.037169 | 0.028 |
| **Residuals** | 40 | 5.556451 | 0.138911 | NaN | 0.962831 | NaN |
| **Total** | 41 | 5.770952 | NaN | NaN | 1 | NaN |
|  |  |  |  |  |  |  |
| Ss+PreT and Ss+PosT Weighted-Unifrac | | | | |  |  |
|  | **Df** | **SumsOfSqs** | **MeanSqs** | **F.Model** | **R2** | **Pr(>F)** |
| **APC** | 1 | 0.008785 | 0.008785 | 0.291111 | 0.015915 | 0.986 |
| **Residuals** | 18 | 0.543208 | 0.030178 | NaN | 0.984085 | NaN |
| **Total** | 19 | 0.551993 | NaN | NaN | 1 | NaN |
|  |  |  |  |  |  |  |
|  |  |  |  |  |  |  |
| Ss+PreT and Ss+PostT UnWeighted-Unifrac | | | | |  |  |
|  | **Df** | **SumsOfSqs** | **MeanSqs** | **F.Model** | **R2** | **Pr(>F)** |
| **Group** | 1 | 0.202403 | 0.202403 | 1.073173 | 0.056266 | 0.341 |
| **Residuals** | 18 | 3.394836 | 0.188602 | NaN | 0.943734 | NaN |
| **Total** | 19 | 3.597239 | NaN | NaN | 1 | NaN |


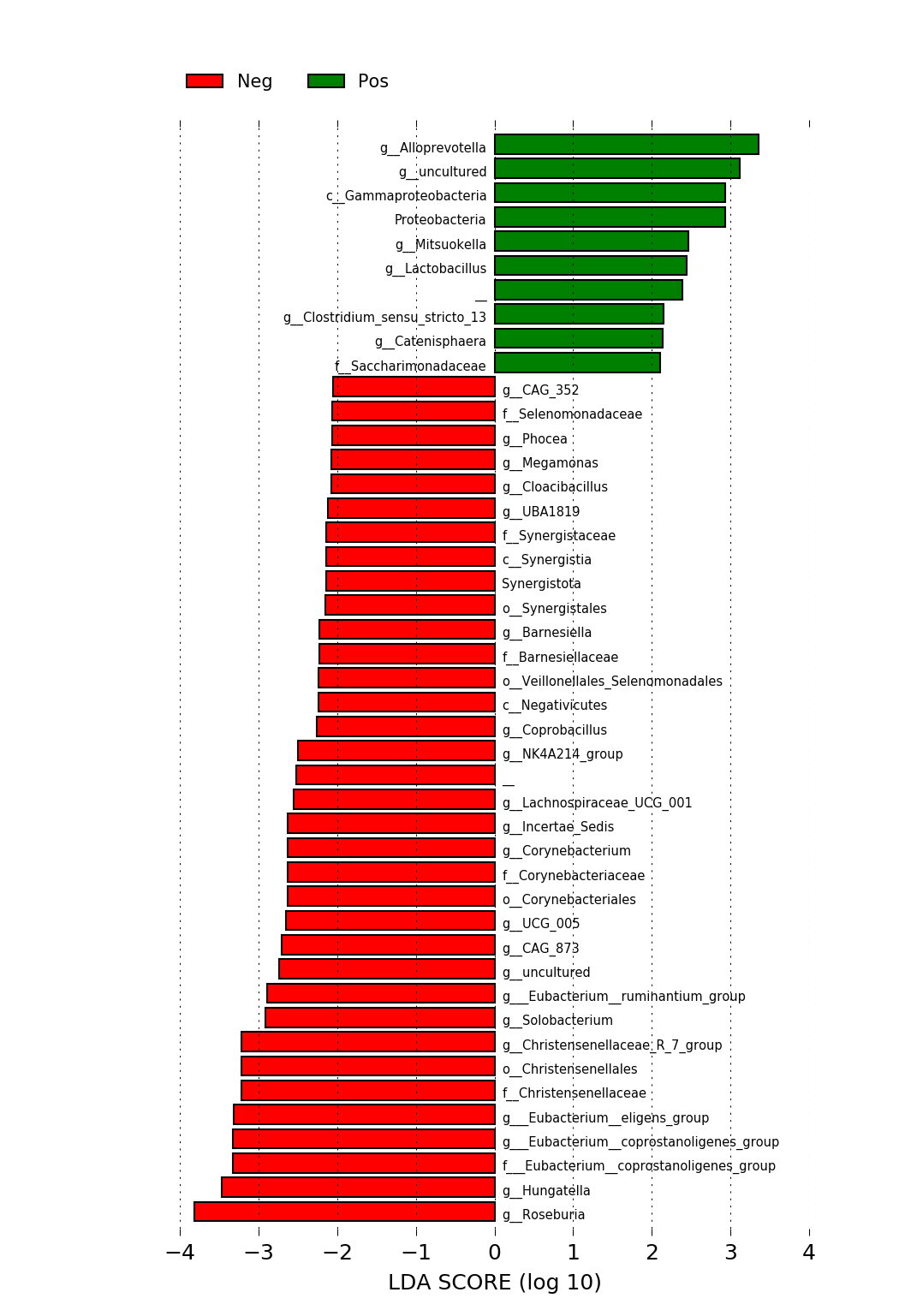


**Supplementary Figure S2**. Histograms of linear discriminant analysis (LDA) effect size (LEfSe) comparison between stool microbiota at the genus level between Pos and Neg at T0.


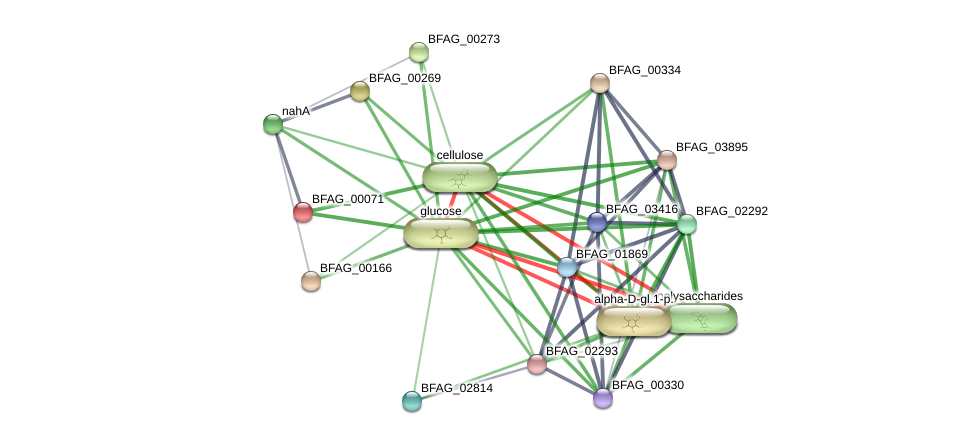


**Supplementary Figure S3**. STITCH analysis of highly expressed proteins in *Bacteroidaceae*, which function in carbohydrate metabolism. *Bacteroides fragilis 3112* was used as the bacteria template to generate the figure.

**Supplementary Table S4.** List of putative proteins of *Bacteroidaceae* in Fig S3**.**

| **Protein Name** | **Gene names** | **Organism** | **Label** |
| --- | --- | --- | --- |
| Glycoside hydrolase family 2 | F2Y53_09670 F2Y70_03640 | *Bacteroides cellulosilyticus* | *BFAG_00269* |
| Glycoside hydrolase | DWX97_00640 | *Bacteroides cellulosilyticus* | *BFAG_00273* |
| Beta-glucosidase | DXB57_15055 | *Bacteroides fragilis* | *BFAG_00166* |
| Tyrosine protein kinase | B5F91_12010 | *Bacteroides sp. An322* | *BFAG_02814 -* |
| Beta-galactosidase | BN604_01537 | Bacteroides intestinalis CAG:315 | *BFAG_00071* |
| Alpha-glucan phosphorylase | C801_03133 | *Bacteroides uniformis dnLKV2* | *BFAG_02292* |
| Beta-N-acetylhexosaminidase, EC 3.2.1.52 |  | uncultured *Bacteroides sp* | nahA |
